# Effects of Rural Restaurants’ Outdoor Dining Environment Dimensions on Customers’ Satisfaction: A Consumer Perspective

**DOI:** 10.3390/foods10092172

**Published:** 2021-09-13

**Authors:** Mian Yang, Shixian Luo

**Affiliations:** 1 Art College, Sichuan Tourism University, Chengdu 610100, China; yangmiansctu@126.com; 2 Graduate School of Horticulture, Chiba University, Chiba 271-8510, Japan

**Keywords:** outdoor dining environment, rural restaurant, satisfaction, consumer perspective

## Abstract

The catering industry is one of the important industries that promote rural tourism development. Hence, rural restaurants have high research value. However, few studies have examined rural restaurants and their outdoor dining environments (ODE). In this study, from the perspective of consumers and using exploratory factor analysis, three ODE dimensions (quality and facilities, image and atmosphere, and landscape elements) were proposed that affect customers’ satisfaction with rural restaurants. Moreover, the differences between different customer groups in terms of the various dimensions were analyzed. The research results provide management recommendations and fundamental knowledge for rural restaurant managers and rural restaurant designers and articulate different consumer groups’ expectations with regard to rural restaurants.

## 1. Introduction

The development of the catering industry must focus on the market perspective [1]. A restaurant’s actual environment and service quality play an important role in influencing consumers’ decisions to visit or refrain from visiting [2,3]. Moreover, creating and maintaining a distinctive atmosphere is considered a key factor for attracting and satisfying consumers [4]. Tourism has been widely developed in global agricultural areas [5]. Because of the introduction of participants (i.e., tourists) with different needs, the protection and sustainable development of agricultural systems is now facing new challenges [6,7,8]. To date, gastronomy tourism has been growing rapidly in popularity in Asia [9], but most studies in this area have focused more on developed countries rather than on developing countries [10]. Restaurants help to revitalize rural economies because they often promote rural development and improvement, consequently stimulating tourism [11]. As a result, we believe that studying the rural dining environment and service quality from the consumer’s perspective can promote the sustainable development of rural gastronomy tourism.

**Figure 1:**
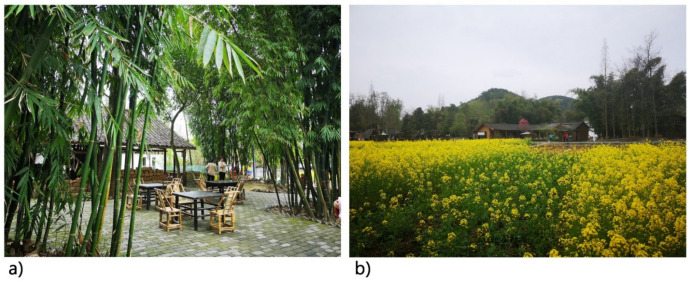
Chinese agritainment: (**a**) The courtyard of a residential area that has been converted into an outdoor dining area; (**b**) An agritainment facility in a rural area is surrounded by natural elements including crops and woods.

The development of rural tourism destinations in China has generally occurred around Chinese agritainment, and agricultural landscapes and lifestyles have functioned as the main attractions (For example, see the Nongjiale, a kind of rural restaurant with local characteristics, which mainly uses the courtyard of the residential area as a dining area (Figure 1) [12]. Rural outdoor dining and recreational activities, which form important features of agritainment tourism, are widely favored by Chinese tourists. As the origin of agritainment as a concept in China, Chengdu, a tourist-oriented city, is well-known as a rural tourism center [13]. Furthermore, Chengdu’s tourism development principle reads “Take tourism as an important industry and build it into an international tourist city” [14].

However, most agritainment facilities in Chengdu are highly homogenized, and less attention has been paid to the design and management of outdoor dining environments (ODE) in rural restaurants [13]. Furthermore, no research has examined or discussed rural restaurants with ODE. Therefore, an ODE study provides valuable insights for managing rural restaurants, especially for developing agritainment in Chengdu. The courtyard functions as the traditional living and activity space of rural dwellings in Chengdu. Exploring its importance and influence as a spatial environment for tourism can improve tourism competitiveness and raise public awareness about agricultural heritage protection [6,7,15].

People from different cultural backgrounds and countries may hold differing views regarding restaurants. For example, Oh et al. [16] found that, while American customers valued food quality, Korean customers focused on the physical environment. This study examines ODE in Chengdu’s rural restaurants and combines the concept of DINESCAPE [17] to explore the evaluation dimensions of rural restaurants’ ODE. These dimensions are critical because they may affect consumer satisfaction regarding rural restaurants. This study’s research objectives were as follows:

(1) Explore ODE dimensions that affect consumers’ satisfaction with rural restaurants;
(2) Explain three ODE dimensions that affect consumer satisfaction considering respondents characteristics.

**Figure 2:**
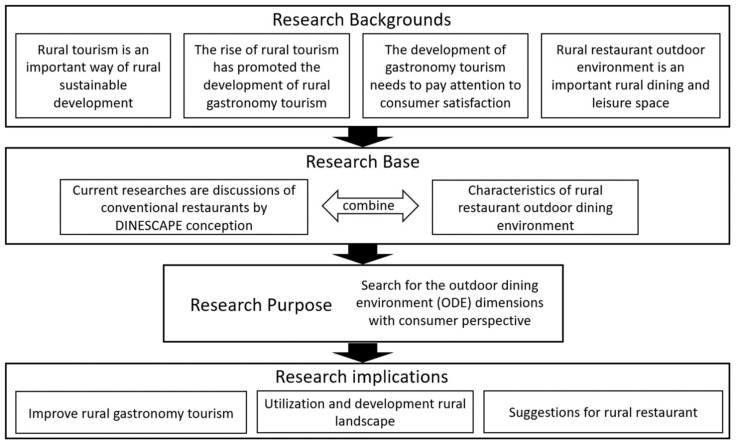
Research framework of the study.

This study aims to (1) provide suggestions for improving ODE management in rural restaurants and (2) discover different consumer groups’ expectations regarding ODE in rural restaurants. Figure 2 illustrates the research framework of this study.

## 2. Literature Review

### 2.1. Rural Restaurant

With increasing urban interest in rural areas, some former rural production landscapes have become urban residents’ consumption and entertainment landscapes [18]. Thus, rural place branding may represent a crucial strategy for coordinating and fascinating this target consumer group [19]. Simultaneously, from an ecological, social, and cultural perspective, protecting agricultural systems is essential for maintaining regional and global sustainability [6,7,20,21]. Thus, integrating agricultural systems’ diversity and evolving values with tourism is essential for supporting agricultural protection and farmers’ livelihoods [8,22]. This protection and development of agricultural systems, rather than remaining static, must reflect dynamic human relationships [6,7]. Regarding gastronomic tourism, food, environment, and novelty value form the main prerequisites for attracting consumers in the promotion of co-development between urban and rural areas [23,24]. Indeed “the roots of gastronomy tourism lie in agriculture, culture, and tourism” [25], hence local agriculture can be used to restore regional characteristics and culture and strengthen local features and attractiveness.

Previous studies have proved that, in some areas, rural space is an important factor for maintaining natural facilities, stimulating outdoor entertainment and tourism, and creating economic activities [26,27]. Albright et al. [28] found that, with regard to restaurants, women and the elderly tended to be more interested in making healthy choices. Bai et al. [29] also found that women were more finicky about selecting safe restaurant environments to protect themselves. Baker et al. [30] compared the importance customers placed on standard restaurant wait staff behaviors and time standards in urban and rural restaurants and found that they could be the key to customer satisfaction and patronage. Eimermann et al. [31] found that rural restaurants provided employment opportunities for immigrants and helped to revitalize rural economies to a certain extent. Rinaldi [32] studied the local characteristics and attractiveness of rural areas and agriculture and proposed that local catering resources must resolve and strengthen the connection between place (territory/geographical dimension) and people (cultural dimension).

In summary, rural restaurants are essential to promote rural economies and protect local cultural capital while also promoting co-development between urban and rural areas. However, research in this area is scarce, and current related work is mainly devoted to restaurant food selection, restaurant indoor environment satisfaction, and sustainable economic development [33]. As areas for “viewing”, enjoying outdoor leisure activities, and dining, rural residential dwellings’ courtyards can function as important environments, which could attract consumers to engage in activities in the attached rural restaurants. However, no study has examined the relationship between ODE and consumer satisfaction thus far.

### 2.2. Rural and Gastronomic Tourism

As a means of promoting a region’s icon/agricultural products, rural gastronomy meets the specific needs of various types of rural tourists [34]. Thus, gastronomic tourism is rapidly developing as an important part of the attractiveness of tourist destinations [35], which is analyzed through tourist behavior. Moreover, the study of gourmet tourism experiences can add value to tourism activities and attract a wide range of potential consumers [36].

Gastronomic and culinary experiences have been recognized as aspects of tourist experiences in which, eating well and the perception of authenticity have become key elements in the construction of memories of the journey [37]. The gastronomic experience is highly subjective [38], for it is a major factor in tourists’ decisions to visit a certain location as well as in their satisfaction with their overall tourism experience [39]. The study of tourists’ gastronomic experiences contributes not only to the understanding of the way in which individual experience processes are constructed, but also to the construction of tourism awareness and the appropriateness of the consumption space from the tourists’ perspective [40]. Hendijani (2016) [41] states that the gastronomic experience includes: heritage, service, gastronomic environment, diversity, availability, sensory, and ingredients. Fields (2002) [1] argues that gastronomic tourism motivation has a cultural character: the desire to discover the destination and its heritage through gastronomy. Ramírez-Gutiérrez et al. [40] confirm that the gastronomic environment has the same importance as the gastronomic product in gastronomic tourism. Bertan [42] suggests that gastronomic tourism analysis should also include visits to restaurants. Various studies show that the research environment is particularly important for gastronomic tourism. However, few studies have addressed these experiential spaces [43].

Currently, researchers are seeking to understand sustainable forms of marketing, competitive positioning, and attracting tourists to gastronomic tourism destinations [44]. Tourism managers are also seeking innovative strategies and competitive advantages to increase repeated visits and recommendations from consumers [45]. In conjunction with the above, we believe that studying the outdoor dining environment of rural restaurants can help to improve regional gastronomic tourism.

### 2.3. Rural Landscape and Consumers Preferences

Human-nature interactions mediated by agriculture result in a diversified rural landscape [46]. The rural landscape is of great value to rural development, as rural tourism is strictly linked to its surroundings and landscape [47]. Devesa et al. [48] indicates that the attributes, leisure infrastructure, and cultural or natural features of the chosen destination are important pull factors for rural tourism. According to several studies, traditional rural landscapes offer rural areas their uniqueness as a result of their historical and cultural values and beauty [49], and in turn this uniqueness influences customers’ tourist destination choices [50]. Rural landscapes and environments, according to Gao et al. [51] are significant in rural tourism planning since tourists are likely to interact with them directly [52]. To improve consumer satisfaction, it is therefore crucial to understand the process of consumer perception and interaction with these landscape features [47].

There have been many studies discussing the public’s perceptions and preferences for rural landscapes. Lindemann-Matthies et al. [53] find that Swiss residents prefer landscapes covered with low-intensively managed and species-rich grasslands, while ecological compensation areas were described as boring despite their productivity. Cong et al. [54] assess the value of recreation in rural landscapes and report that visitors are willing to pay a higher price for high-quality landscape elements. Yao et al. [55] investigate how the public rate the visual quality of rural landscapes and find that vegetation and water are major indicators of visual quality.

Consumer preference refers to how much consumers like a product. It has been suggested that aesthetically pleasing landscapes are more likely to be appreciated [56]. Understanding public preferences is important for regional policy decisions as well as landscape development and use. In sum, this study argues that landscape and consumer preferences are inextricably intertwined, with customer preferences for rural landscape elements potentially influencing consumer satisfaction [57].

### 2.4. Dining Environment Research

A given restaurant’s physical environment is often the first factor a consumer perceives after entering the establishment. This key factor attracts and satisfies customers and improves financial performance by maximizing restaurant revenue and market share in the long term [4,33,58,59,60]. The influence of restaurants physical environment on customer behavior has been studied by academics in various countries [17,61,62,63,64]. Although some esthetic studies have examined hotel environments [65], few studies have focused on restaurant esthetics. Moreover, little consideration has been paid to the overall esthetic experience, which influences customers dining experiences [66]. In contemporary times, design involves esthetic quality as well as esthetic perception [67]. Experienced designers focus on the beauty of intangible and tangible things and observers’ feelings. Therefore, esthetic objects and esthetic subjects should be considered together.

Ryu and Jang [17] proposed the concept of DINESCAPE. This concept regarding man-made physical and human environments in restaurant dining areas includes six dimensions: facility esthetics, ambience, lighting, table setting, layout, and service personnel. Scholars have also studied the association between consumer dining experience and individual factors, such as facility esthetics [68], lighting [69], physical environment [64], layout [70], table setting [71], and service personnel [17]. Using Ryu and Jang’s research, Horng and Hsu [72] summarized restaurant landscape environments into four dimensions: the physical environment (including architecture, restaurant name, signage, interior design and decoration, furniture and equipment, layout, lighting, temperature, aroma, and music), products and services (including the appearance and flavor of food and beverages, menu items and design, tableware, employee expressions, employee body movements and gestures, employee introduction, communication, and storytelling), employees’ esthetic characteristics (including employees’ appearance, voice, and body odor), and other customers’ esthetic characteristics (including customers’ appearance, voice, behavior, and etiquette). Most previous studies on dining environments have focused on architecture and indoor environments. Few studies have concentrated on ODE in restaurants and their impact on consumer experience. Riley [73] and Canny [74] showed that the physical environment could positively affect customer satisfaction and that customer satisfaction, in turn, could positively affect behavioral intentions. Since physical environment is a major influencing factor for consumer response in hedonic services, it is necessary to understand how consumer satisfaction and behavior can change depending on consumers’ perception of physical environmental factors [63]. Therefore, this study utilized consumer “satisfaction” for assessing consumers’ willingness to spend and their intention to visit. Table 1 shows the list of studies related to the six dimensions of DINESCAPE.

#### 2.4.1. Facility Aesthetics

Facility esthetics refers to architectural design, interior design, and decoration, which are essential factors for attracting and retaining restaurant customers [68]. For example, the color scheme of decorative walls and floor coverings, furniture, pictures/paintings, plants/flowers, wall decorations, and so on are important influencers of customer pleasure, arousal, and behavioral intentions [75]. Customers may be affected by the restaurant’s color scheme, and different colors could stimulate different emotions, feelings, or emotional associations [76]. Therefore, many restaurant managers recognize and use facility esthetics to create specific restaurant themes [75]. The study by Horng and Hsu [72] classified influencing factors of facility aesthetics into buildings, restaurant names, signage, interior design, decoration, furniture, and equipment.

#### 2.4.2. Ambience

Ambience refers to a distinct set of factors [30] that affect non-visual senses and may have subconscious effects on consumers; this includes music, smell, and temperature. Previous studies have found that lively music can (1) influence customers’ perceptions of commercial places [77]; (2) elicit emotions [64]; (3) affect customer satisfaction and relaxation [78]; (4) increase shopping time and waiting time [79]; (5) reduce perceived shopping time and waiting time [79]; (6) affect dining speed [80]; and (7) affect purchase intention [30]. On the contrary, noise and music can also affect restaurant customers’ mood. Customers may spend lesser time in a given restaurant if its music or environment noise is loud, fast, or disturbing [81]. Furthermore, retail industry research has attested to the powerful influence of pleasant smells for increasing sales [82]. A scent can affect consumers’ mood, emotions, or purchase intention [83].

#### 2.4.3. Lighting

Research has shown that lighting levels are correlated to individual emotional responses. Ragneskog et al. [69] showed that lighting level preferences could impact personal emotional responses. Ryu and Han [63] suggested that respondents may experience a more positive impact under low light conditions and that lighting can function as a major significant physical stimulus in restaurants. The bright lighting of fast-food restaurants (e.g., McDonald’s) could signal fast service and relatively low prices, while some businesses’ soft and warm lighting could signal a more comprehensive range of services and high prices. Lin et al. [71] suggested that bright lighting may reduce the amount of time customers spend in restaurants, while soft or warm lighting (including candlelight) could entice consumers to stay longer and enjoy unplanned desserts or extra drinks.

#### 2.4.4. Layout

Layout refers to how objects (such as machinery, equipment, and furniture) are arranged within a given environment [76]. Past research has shown that narrow layouts directly affect consumers’ quality perception and excitement and indirectly affect their desire to return to a given establishment [68]. Striking or unusual layouts can promote satisfaction, pleasure, or hedonic needs among consumers [68]. Restaurant management must note and understand where returning customers wish to sit and how they want to move through the restaurant [63].

#### 2.4.5. Table Settings and Placements

Table settings and placements can significantly affect overall customer experience. From an overall view of restaurant management, table placement can convey a sense of privacy, describe required functions for customers, and serve as boundary setters for consumers [71]. From a detailed view of restaurant management, high-quality tableware, glassware, and tablecloths can all effectively influence customers’ perceptions regarding the restaurant’s overall service quality. Table decorations (including attractive candles and flowers) can also positively influence customers’ feelings (e.g., make them believe they are in a prestigious environment) [63]. Restaurant management should thus focus on cultivating customers’ perceptions regarding table setting. For upper-class customers, this is an essential determinant for perceiving a prestigious image of—and retaining their patronage at—upscale restaurants.

#### 2.4.6. Service Personnel

The term “service personnel” refers to employees in service environments [17]. The influencing factors related to this dimension include employee appearance, number of employees, and gender of employees. For example, Ryu and Jang found that professional employee uniforms effectively conveyed the image and core values of the organization in a very close and personal way [17]. Baker [30] suggested that the number and appearance of employees in a given establishment could be associated with influences on its customers’ positive emotions.

## 3. Methodology

### 3.1. Questionnaire Design

The questionnaire contained three parts. The first part involved items related to respondents’ sociodemographic characteristics (gender, age, place of residence, whether they were local residents, monthly income, education, and job). Two additional topics were included: “frequency of dining at restaurants (weekly)” and “frequency of dining at rural restaurants (yearly).” The second part contained items related to restaurant preferences (including three items: “the purpose of choosing a country restaurant”, multiple choice; “the criteria for the choice”, multiple choice; and “preferred restaurant-style”, single choice). In the third part, we used Ryu and Jang’s [17] DINESCAPE concept and the four dimensions proposed by Horng [72] as theoretical knowledge bases and combined them with the distinct landscape characteristics of Chengdu’s rural areas to present items related to our ODE research framework. This framework is designed to examine the impact of various environmental dimensions of ODE on consumers’ satisfaction with rural restaurants.

Furthermore, we consulted two experts in landscape design, two experts in catering environment design, five experts in tourism, one rural restaurant manager, and ten consumers to ensure that the questionnaire met our research purposes. Based on their suggestions, we adjusted the questionnaire and determined its final version (containing 21 items). Respondents were asked to use a 7-point Likert scale (1 = no effect at all, 7 = very large effect) to score each ODE item. To ensure that interviewees had a clearer understanding of each item, we added photos to explain each item (the photos were all taken by the authors).

### 3.2. Data Collection

**Figure 3:**
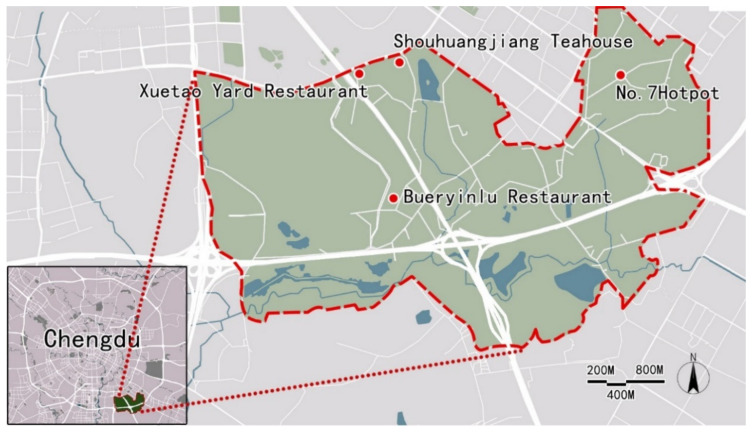
Study area and the four selected rural restaurants.

Completed questionnaires were collected from four rural restaurants in Sanshengxiang, Chengdu, China, between March and May 2021 (Figure 3). Sanshengxiang was selected as the research site because it is a famous rural tourism area in Chengdu and it houses many different styles of rural restaurants. Second, four restaurants were selected as this study’s main questionnaire collection locations based on diners’ online evaluation of their services. Based on their Dazhongdianping ratings (the most widely used catering forum in China, http://www.dianping.com/, accessed on: 20 February 2021), four of the most popular (highest overall rating in the site) and distinctive rural restaurants in the region were selected: Bueryinlu Restaurant (a rural style underground restaurant), No. 7 Hotpot (Sichuan hotpot), Shouhuangjiang Teahouse (tea and gastronomy), and Xuetao Yard Restaurant (Sichuan cuisine).

This study implemented a convenience sampling method to collect the questionnaires. Research assistants randomly contacted customers around these rural restaurants and provided them with the questionnaires. These customers’ companions were also encouraged to participate. Although this study used a non-probability sampling method, this method has been widely used in social science studies [84] and has proven reliability [29]. All participants were informed about the study’s purpose and the survey’s anonymity to ensure the quality of the questionnaire, and each participant’s oral consent was obtained. The research assistants remained in the restaurant area to answer the interviewees’ questions. These research assistants were all students of Sichuan Tourism University, and they were all trained in survey techniques and knew the purpose of the survey. In all, 488 questionnaires were obtained. Among these, 12 samples were obtained from the group of junior high schoolers and lower, and they were therefore excluded because this sample size was too small to be representative. Finally, 476 valid questionnaires were obtained and an effective rate of 97.5% was reached. The necessary ethical approvals were obtained from the Academic Committee of Sichuan Tourism University.

### 3.3. Data Collection

SPSS (version 20.0; IBM Corp., Armonk, NY, USA) and Microsoft Excel were used to analyze the survey data. The average, frequency, and percentage were calculated based on each response. Exploratory factor analysis (EFA) was performed to determine the main dimensions affecting rural restaurant satisfaction, and Cronbach’s alpha reliability was calculated [85]. Furthermore, the average score and standard deviation of each dimension were calculated. The Shapiro–Wilk test was used for assessing the normality of each item and nonparametric tests, namely the Mann–Whitney U test and the Kruskal–Wallis H test, were used for determining the differences between groups. A result having a *p*-value of < 0.05 was considered statistically significant.

## 4. Results

### 4.1. Basic Information

Among the 476 respondents, 178 (37.4%) were male, and 298 (62.6%) were female. There were 187 people (39.3%) aged between 31 and 40 years and 148 (31.1%) people aged over 41 years. Furthermore, 239 people (50.2%) were local residents of Chengdu and 291 (61.1%) lived in urban areas. Education-wise, 420 (88.2%) respondents had a university degree or a higher qualification. Finance-wise, 167 (56.1%) had a monthly income of more than 4500 RMB (the average wage in Chengdu in 2019 is 4488 RMB; http://cdstats.chengdu.gov.cn/ accessed on: 29 August 2021). A total of 224 people (47.1%) dined at the restaurant 1–3 times a week (Table 2).

### 4.2. Restaurant Preference-Related Results

**Figure 4:**
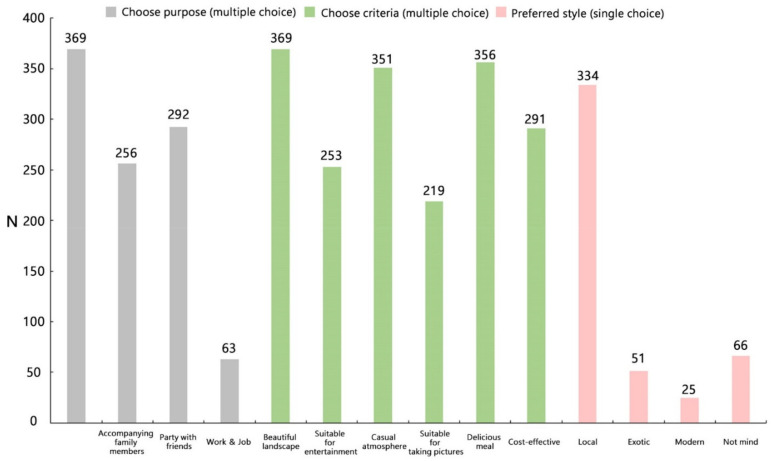
Preferences for rural restaurant choice.

Figure 4 shows that “Relaxing & recreation” formed the main purpose of choosing a rural restaurant (369). Other reasons included “Accompanying family members” (256) and “Partying with friends” (292). Relatively few people chose rural restaurants because of “Work & job” (63). In terms of the selection criteria for rural restaurants, “Beautiful landscape” (369), “Delicious meal” (356), and “Casual atmosphere” (351) were the three main criteria. Other preference criteria also included “Cost-effective” (291), “Suitable for entertainment” (253), and “Suitable for taking pictures” (219). Furthermore, 334 people (70.2%) were more inclined to dine in an agritainment-related establishment with local characteristics.

### 4.3. Exploratory Factor Analysis

EFA using principal component analysis with varimax rotation was conducted to identify the main dimensions affecting customers’ satisfaction with rural restaurants. Previous research showed that each factor can only be classified under one dimension at a time and that the load should exceed 0.5 [86]. Thus, four items (plant aroma, tree, agricultural landscape, and waterscape) were removed because their loads in two dimensions were >0.5 (Table 3). The smallest normalized alpha coefficient (0.918) indicated that the data were ideal because all the relevant coefficients were greater than 0.7 [87]. The Kaiser–Meyer–Olkin value was 0.951 (*p* < 0.001), indicating that the results of the factor analysis were reliable.

The principal component analysis results showed that the items affecting customers’ satisfaction with rural restaurants could be divided into three dimensions: quality and facilities (uniform, appearance, garnish, table setting, service quality, table placement, illumination, and decorations), image and atmosphere (name, natural sound, signage, and music), and landscape elements (pavement, artificial structure, buildings, and ornamental plants). The cumulative explanatory variance of the three factors was 70.630%, and “Quality and facilities” accounted for the largest proportion (57.769%). Furthermore, the average variance extracted (AVE) and composite reliability (CR) were calculated to determine the appropriateness of the factor analysis. The calculation formula was as follows:

AVE = (Σλ^2 )/n (1)

CR = (Σλ)^2/((Σλ)^2+Σε) (2)

where λ is the factor loading, n is the number of measurement indexes for the dimension, and ε is the residual variance. Table 4 shows the AVE and CR values of the three dimensions. According to Fornell and Larcker [88], AVE > 0.4 and CR > 0.6 are considered acceptable, and our results indicate that both meet the requirements.

Moreover, Table 3 shows that “landscape elements” had the largest overall impact (M = 4.97) and that each sub-dimension (Pavement, Artificial structure, Buildings, Ornamental plants) impact was close (from 4.93 to 5.03). The “Image and atmosphere” dimension had the most negligible impact (M = 4.29). Of these subsections, “service quality” (M = 5.23), “ornamental plants” (M = 5.03), “buildings” (M = 4.99), “artificial structures” (M = 4.95), “pavement” (M = 4.93), and “illumination” (M = 4.92) all had a relatively high impact on customers’ satisfaction with rural restaurants. In contrast, the restaurant name (M = 3.91) and signage (M = 4.16) were evaluated as having the least impact.

### 4.4. Relationships between Demographic Characteristics and the Three Dimensions

Table 5 lists the average scores of each subsection as well as the significant differences based on the Mann–Whitney U test (two groups)/Kruskal–Wallis H test (greater than two groups). In general, the subsections of each dimension were different because the interviewees had different subgroups (gender, age, resident status, monthly income, education level, and frequency of dining at restaurants weekly). Specifically, in terms of gender, women (4.93) were more affected by quality and facilities compared to men (4.57). In addition, women scored higher than men on the other two dimensions, but neither was significant. Thus, female participants were considered to be most vulnerable to the ODE dimension. For people in different age groups, people aged over 50 years (3.67) were less likely to be affected by the image and atmosphere dimensions. The other two ODE dimensions showed no significant variation among respondents of all ages. Local residents were more likely to be affected by the three dimensions than non-locals. Despite the fact that respondents in suburban regions believed these ODE dimensions had a bigger impact on satisfaction, none of the three factors showed a difference according to the customers’ place of residence (i.e., rural, urban, suburban). In terms of monthly income, all three dimensions showed two extremes; that is, people with a monthly income of less than 2,000 RMB and more than 8000 RMB had higher scores and showed significant differences compared to the middle-income group. Moreover, in terms of their satisfaction with rural restaurants, people with higher education levels and a greater frequency of dining at the selected restaurants each week tended to be more easily affected by these three dimensions. In contrast, type of work and frequency of dining in rural restaurants did not show any significant differences in all the dimensions.

## 5. Discussion and Implications

This study examined ODE in Chengdu’s rural restaurants and based on the concept of DINESCAPE [17] to explore appropriate evaluation dimensions for ODE in rural restaurants. These dimensions could affect consumers’ satisfaction with rural restaurants. Based on existing knowledge, this research combined selected landscape characteristics of rural outdoor environments to add more items that could impact customers’ satisfaction with rural restaurants. Furthermore, using EFA, three ODE dimensions that could affect satisfaction were proposed: quality and facilities (uniform, appearance, garnish, table setting, service quality, table placement, illumination, and decorations), image and atmosphere (name, natural sound, signage, and music), and landscape elements (pavement, artificial structure, buildings, and ornamental plants). Unlike previous DINESCAPE studies, this study focused on rural restaurants and ODE. Furthermore, the principal component analysis results (Table 3) indicated that consumers were highly concerned about ODE landscape elements as well as restaurant quality. This result could be explained by the fact that tourists in rural areas are often more inclined to experience natural landscapes. Our current result is of significance because it is the first study to measure the dimensions of consumer perceptions of the outdoor dining environment in rural restaurants.

Of the three ODE dimensions, consumers are most concerned with the landscape element dimension, followed by quality and facilities, especially among high-income consumers who likes to dine out often. These people are often potential or actual return customers, and they are believed to provide a more accurate evaluation of rural restaurants [89]. This is similar to the previous result that the most critical motivation for consumers to visit the rural areas is the high quality of the traditional rural landscape [47]. Among the sub-dimensions, some items are similar to DINSCAPE with a high impact, such as service quality and light. This result indicates that service quality and lighting of the restaurant are both important dimensions that affect the consumer experience, whether it is the interior or the outdoor environment of the restaurant. Furthermore, music and natural sounds receive relatively high ratings in the image and atmosphere dimensions, which is consistent with a study by Liu et al. [90] but differs from the observations of Horng [72], with no significant effect of signage and restaurant names found in our study. In addition, Sarmiento [91] argues that female consumers have a higher green propensity index than men, but willingness to pay is not a good indicator of a consumer’s green propensity as a proxy. Therefore, in our study, we expand the knowledge in this area through consumer perceptions of the rural restaurants ODE dimension.

Rural restaurant managers should thus focus on factors that influence their dining satisfaction [63] and should consider investing more funds in rural outdoor landscape elements to improve customer dining experience. Furthermore, the findings of the ODE dimension study can be used by restaurant managers to examine the status of the restaurant’s ODE and compare it to that of other restaurants. Managers planning renovations or redecorating should perform a satisfaction survey to generate relevant business strategies. Restaurants can create a leisurely and beautiful traditional agricultural landscape to provide consumers with an outdoor dining environment for recreation and photo-taking, thus improving the overall consumption level and consumer satisfaction of rural restaurants, in conjunction with respondents’ “purpose of choosing a restaurant.” Besides, restaurant managers must effectively conduct preliminary business-ability training for potential service personnel to impart professional skills, such as improving service quality, adopting uniform dressing, and minding professional appearance.

Understanding the behavioral demands of different populations when it comes to outdoor recreation is becoming increasingly important [92]. Managers of rural restaurants should devise strategies and experience packages to appeal to different customers. For example, according to the results in Figure 4, local consumers were more inclined toward the local style environment. This finding differed from that of Xue et al. [93], who suggested that residents were not interested in local restaurants and preferred exotic restaurants. Managers should determine the style of the restaurant based on the primary source of visitors, rather than deliberately creating an exotic atmosphere. Women and elderly individuals (>50 years old) were more finicky about restaurant quality and facilities. The results in this regard were consistent with those of Bai et al. [29]. In their study, women usually focus on some additional indicators to avoid unsafe restaurants and any related danger. Other factors could include the fact that these people are more likely to be primary carers for their children [94]. As a result, managers of restaurants with a predominantly female/elderly consumer base should focus on restaurant quality, greenness, and child safety as the focus of the restaurant environment. Pan et al. [95] advocate short-term and frequent travel strategies to improve elderly people’s travel satisfaction and overall quality of life through good products and services since expenses are no longer a limiting condition affecting their inclination to travel. Therefore, restaurant managers can improve elderly people’s dining experiences by improving restaurant quality. Furthermore, rich landscape elements can be added to the restaurant environment to meet their needs for leisure/natural experiences, thereby increasing their willingness to revisit. Moreover, our findings show a U-shaped tendency across persons of various income levels about their ODE perceptions. Those with a household income of less than 2000 RMB and those with a household income of more than 8000 RMB both agree that the three characteristics have a great impact on satisfaction. The findings imply that low-income people seek value for money, whereas high-income persons appear to seek out a high-quality dining experience on purpose. Finally, the effect of the three ODE dimensions on satisfaction increases significantly with education, which can be explained by the fact that people with higher education are more concerned about the dining environment. Finally, the effect of the three ODE dimensions on satisfaction increases significantly with education, indicating that people with higher levels of education care more about the dining environment. In summary, managers of rural restaurants need to focus on the impact of these dimensions and design ODE to improve consumer satisfaction according to different target groups.

Moreover, for the tourism policy aspect. It has been recognized that gastronomy tourism discourse also includes ethical and sustainable values based on local dimensions, such as territory, landscape, culture, and local products [96]. Traditional rural landscapes are highly distinctive and enhance the tourism brand of a region [49,97]. This article recommends that planners and managers attempt to integrate tourism and landscape architecture by supporting agricultural differentiation, maintaining local resources (biodiversity and natural and cultural resources), and exploring ODE dimensions for rural restaurants. This study’s results could thus promote the development of rural tourism and protect rural landscapes and local cultures. Given that many rural restaurants are self-employed [98], the local government can provide relevant training to local farmers and promote the importance of rural landscape environments for retaining the original local rural features. Furthermore, the present Chengdu Nongjiale is homogenized and follows a single tourism concept. Sanshengxiang is a flower-based rural tourism destination, so it may use its natural resources to build and develop some rural theme restaurants to attract visitors and differentiate rural gastronomic tourism. Visitors to Sanshengxiang value the rural ecological environment and the quality of the rural landscapes, according to a previous study [54]. However, as urbanization progresses, rural landscapes are becoming increasingly scarce. As a result, ODE in rural restaurants can provide tourists with a pleasant and authentic rural landscape setting while also promoting environmental protection and sustainable tourism development.

This study had some limitations. First, the study area was limited to the Sanshengxiang rural area of Chengdu. Thus, this study’s results could have some bias and lack generalizability if applied to other countries/regions. Future research should be conducted in different regions to test the applicability and generalizability of the three ODE dimensions developed in this study. Second, in this study, consumers in rural restaurants in Chengdu were the research object. Thus, this study’s results should be carefully evaluated before they are applied to consumers from other cultural backgrounds and countries. Furthermore, this study utilized convenience sampling. Therefore, although the interviewee sample included multiple age groups, the participants were not randomly sampled with equal probability, and this could lead to a bias in the results. This is only an exploratory study, and further research on the developed ODE for rural restaurants would be valuable. Finally, this was a cross-sectional study based on questionnaires. Thus, the current results cannot confirm whether the impact of these dimensions will vary with time and season.

Overall, despite these limitations, the findings of this study are still useful. For the first time, this study examined how consumers perceive the outdoor dining environment in rural restaurants to provide guidelines for rural tourism and restaurant development. Second, there is a lack of research on rural restaurants, and the results of this study can provide a reference for research on rural dining in other regions/countries. In addition, from the perspective of consumers, this study presents three elements of rural restaurant ODE. Researchers in various countries/regions can use our methods to test the ODE dimensions of different sorts of restaurants in the future. Further, the integration of rural landscape elements with restaurants could also contribute to the conservation of landscape resources to achieve the goal of sustainable tourism development. Finally, to the best of our knowledge, few surveys on consumer perceptions of rural restaurants have been performed. As a consequence, despite the fact that we only conducted a sample survey in Chengdu, the study data are significant for consumer behavior research. We encourage additional research to expand the applicability of this study.

## Figures and Tables

**Table 1:** List of studies related to the six dimensions of DINESCAPE.

Dimensions	Features	Attributes	Citations
Facility aesthetics	Buildings	Attracting and retaining restaurant customers; influence customer pleasure, arousal, and behavioral intentions	[68,72,75,76]
	Restaurant names		
	Signage		
	Interior design		
	Decoration		
	Furniture		
	Equipment		
	Plants/Flowers		
Ambience	Music	Elicit emotions; affect customer satisfaction, relaxation, dining speed, and purchase intention	[30,64,77,78,79,80,81,82,83]
	Scent		
	Smell		
	Temperature		
Lighting	Lighting	Impact personal emotional responses and entices consumers to stay longer	[63,69,71,72]
	Candlelight		
Layout	Objects layout	Affect consumers’ quality perception, desire to return; promote satisfaction, pleasure, or hedonic needs	[63,68,72,76]
Table settings and placements	Table placement	Affect overall customer experience; serve as boundary setters; influence customers’ perceptions regarding the restaurant’s overall service quality	[63,70,71,72]
	Tableware		
	Glassware		
	Tablecloths		
	Table decorations		
Service personnel	Employee appearance	Conveyed the image and core values of the organization; influences customers’ positive emotions	[17,30,72]
	Employee number		
	Employee gender		

**Table 2:** Profile of the samples.

Sociodemographic Characteristics	Category	n.	Percentage (%)
Gender	Male	178	37.4
	Female	298	62.6
Age	18–30	101	21.2
	31–40	187	39.3
	41–50	113	23.7
	>50	75	15.8
Local residents	Yes	239	50.2
	No	237	49.8
Place of residence	Rural	71	14.9
	Urban	291	61.1
	Suburban	114	23.9
Monthly income (RMB)	<2000	87	18.3
	2000–4500	122	25.6
	4500–8000	158	33.2
	>8000	109	22.9
Education	High school	56	11.8
	College	338	71
	Postgraduate	82	17.2
Frequency of dining at restaurants (weekly)	Rarely	106	22.3
	1–3	224	47.1
	3–5	56	11.8
	>5	90	18.9
Frequency of dining at rural restaurants (yearly)	1/week	61	12.8
	1/month	206	43.3
	>1/month	209	43.9
Job	Student	62	13
	Staff	138	29
	Civil servants	118	24.8
	Self–employment	90	18.9
	Retirement	68	14.3

**Table 3:** Results of the factor analysis.

Factor Name and Items	Mean (SD)	Rotated (Varimax) Factors		
		1	2	3
Factor 1: Quality and facilities	**4.79 (1.31)**			
Uniform	4.72 (1.58)	0.763		
Appearance	4.38 (1.63)	0.755		
Garnish	4.63 (1.56)	0.755		
Table setting	4.87 (1.57)	0.727		
Service quality	5.23 (1.55)	0.676		
Table placement	4.77 (1.59)	0.687		
Illumination	4.92 (1.60)	0.643		
Decorations	4.82 (1.57)	0.601		
Factor 2: Image and atmosphere	**4.29 (1.44)**			
Name	3.91 (1.73)		0.737	
Natural sound	4.47 (1.83)		0.733	
Signage	4.16 (1.83)		0.699	
Music	4.62 (1.70)		0.567	
Factor 3: Landscape elements	**4.97 (1.40)**			
Pavement	4.93 (1.61)			0.815
Artificial structure	4.95 (1.58)			0.797
Buildings	4.99 (1.57)			0.715
Ornamental plants	5.03 (1.59)			0.702
Eigenvalue		11.554	1.399	1.174
% of variance		57.769	6.993	5.868
Cumulative %			64.762	70.630
Standardized Cronbach’s α		0.935	0.918	0.923

Note: KMO = 0.951; *p* < 0.001. Four items (plant aroma, tree, agricultural landscape, and waterscape) were removed because their loads in two dimensions were >0.5.

**Table 4:** Appropriateness test for factor analysis.

Factors	CR	AVE
Quality and facilities	0.874	0.499
Image and atmosphere	0.780	0.473
Landscape elements	0.844	0.576

**Table 5:** Relationships between demographic characteristics and the three dimensions (*n* = 476).

Demographic Characteristics		Quality and Facilities	Image and Atmosphere	Landscape Elements
Gender	Male	**4.57 (1.48) ^a^**	4.11 (1.54) ^a^	4.85 (1.51) ^a^
	Female	**4.93 (1.18) ^b^**	4.39 (1.37) ^a^	5.05 (1.32) ^a^
Age	18–30	4.72 (1.40) ^a^	**4.46 (1.46) ^a^**	4.92 (1.47) ^a^
	31–40	4.92 (1.26) ^a^	**4.43 (1.39) ^a^**	5.14 (1.30) ^a^
	41–50	4.74 (1.32) ^a^	**4.31 (1.50) ^a^**	4.90 (1.44) ^a^
	>50	4.66 (1.30) ^a^	**3.67 (1.30) ^b^**	4.74 (1.44) ^a^
Local residents	Yes	**4.99 (1.09) ^a^**	**4.52 (1.26) ^a^**	**5.15 (1.22) ^a^**
	No	**4.59 (1.48) ^b^**	**4.05 (1.57) ^b^**	**4.79 (1.53) ^b^**
Place of residence	Rural	4.66 (1.64) ^a^	4.26 (1.68) ^a^	4.76 (1.69) ^a^
	Urban	4.81 (1.25) ^a^	4.25 (1.41) ^a^	4.98 (1.38) ^a^
	Suburban	4.84 (1.25) ^a^	4.40 (1.38) ^a^	5.09 (1.23) ^a^
Monthly income	<2000	**5.15 (1.14) ^a^**	**4.69 (1.36) ^a^**	**5.30 (1.18) ^a^**
	2000–4500	**4.54 (1.43) ^b^**	**4.16 (1.57) ^b^**	**4.64 (1.61) ^b^**
	4500–8000	**4.72 (1.30) ^b^**	**4.05 (1.43) ^b^**	**4.91 (1.38) ^a^**
	>8000	**4.90 (1.27) ^a^**	**4.46 (1.29) ^a^**	**5.18 (1.25) ^a^**
Education	High school	**4.10 (1.57) ^a^**	**3.58 (1.57) ^a^**	**4.18 (1.69) ^a^**
	College	**4.82 (1.29) ^b^**	**4.31 (1.45) ^b^**	**5.02 (1.37) ^b^**
	Postgraduate	**5.18 (1.03) ^c^**	**4.70 (1.11) ^c^**	**5.34 (1.06) ^b^**
Frequency of dining at restaurants (weekly)	Rarely	**4.72 (1.19) ^a^**	**4.17 (1.36) ^a^**	**4.92 (1.24) ^a^**
	1–3	**4.67 (1.43) ^a^**	**4.22 (1.50) ^a^**	**4.83 (1.52) ^a^**
	3–5	**4.87 (1.07) ^a^**	**4.18 (1.11) ^a^**	**5.04 (1.19) ^a^**
	>5	**5.14 (1.22) ^b^**	**4.68 (1.53) ^b^**	**5.35 (1.31) ^b^**
Frequency of dining at rural restaurants (yearly)	1/week	4.67 (1.75) ^a^	4.19 (1.71) ^a^	4.91 (1.59) ^a^
	1/month	4.77 (1.30) ^a^	4.24 (1.41) ^a^	4.87 (1.44) ^a^
	>1/month	4.85 (1.17) ^a^	4.36 (1.39) ^a^	5.10 (1.28) ^a^
Job	Student	4.89 (1.15) ^a^	4.46 (1.32) ^a^	5.13 (1.28) ^a^
	Staff	4.79 (1.29) ^a^	4.42 (1.46) ^a^	5.06 (1.35) ^a^
	Civil servants	4.78 (1.15) ^a^	4.22 (1.35) ^a^	5.00 (1.24) ^a^
	Self-employment	4.78 (1.63) ^a^	4.28 (1.68) ^a^	4.84 (1.67) ^a^
	Retirement	4.77 (1.32) ^a^	4.00 (1.30) ^a^	4.79 (1.46) ^a^

Note: The numbers in the table indicate the mean value and standard deviations of the subgroups of the demographic characteristics in that dimension; Bold text indicates significant differences; The same letter indicates that the average scores are not significantly different from each other. The significance is compared by column; The significance level of 5% was based on the Mann-Whitney U test (two groups) or Kruskal-Wallis H test (more than two groups).

## Data Availability

The data presented in this study are available on request from the corresponding author. The data are not publicly available due to privacy.
